# Weights and importance in composite indicators: Closing the gap

**DOI:** 10.1016/j.ecolind.2017.03.056

**Published:** 2017-09

**Authors:** William Becker, Michaela Saisana, Paolo Paruolo, Ine Vandecasteele

**Affiliations:** European Commission, Joint Research Centre, Via Enrico Fermi, 2749, 21027 Ispra VA, Italy

**Keywords:** Composite indicators, Sensitivity analysis, Correlation, Splines, Gaussian process, Smoothing, Optimisation

## Abstract

Composite indicators are very popular tools for assessing and ranking countries and institutions in terms of environmental performance, sustainability, and other complex concepts that are not directly measurable. Because of the stakes that come with the media attention of these tools, a word of caution is warranted. One common misconception relates to the effect of the weights assigned to indicators during the aggregation process. This work presents a novel series of tools that allow developers and users of composite indicators to explore effects of these weights. First, the importance of each indicator to the composite is measured by the nonlinear Pearson correlation ratio, estimated by Bayesian Gaussian processes. Second, the effect of each indicator is isolated from that of other indicators using regression analysis, and examined in detail. Finally, an optimisation procedure is proposed which allows weights to be fitted to agree with pre-specified values of importance. These three tools together give developers considerable insight into the effects of weights and suggest possibilities for refining and simplifying the aggregation. The added value of these tools are shown on three case studies: the Resource Governance Index, the Good Country Index, and the Water Retention Index.

## Highlights

• Composite indicators are widely used in sustainable development and elsewhere.
• The effect of weights used in aggregating indicators is complex.
• Three tools are presented which help developers and users to investigate effects of weights.
• Case studies related to sustainable development demonstrate the benefits.

## 1 Introduction

Composite indicators (also known as synthetic indices or performance indices) are popular tools for assessing the performance of countries/entities on human development, sustainability, perceived corruption, innovation, competitiveness, or other complex phenomena that are not directly measurable and not uniquely defined. Examples include the Human Development Index (Jahan, 2015), the Sustainable Society Index (Van de Kerk and Manuel, 2008), the Financial Secrecy Index (Cobham et al., 2013) and the Environmental Performance Index (Hsu, 2016). Composite indicators are employed for many purposes, including policy monitoring, communication to the public, and generating rankings.

The popularity of rankings owes to two main reasons. First, their simplicity: they provide a summary picture of the multiple facets or dimensions of complex, multidimensional phenomena in a way that facilitates evaluation and comparison. Second, rankings force institutions and governments to question their standards; rankings are drivers of behaviour and of change (Kelley and Simmons, 2015). Hence, it comes at no surprise that over the past two decades there has been a turbulent growth of performance indices. Bandura (2011) provides a comprehensive inventory of over 400 country-level indexes monitoring complex phenomena from economic progress to educational quality. Similarly, a more recent inventory by the United Nations (Yang, 2014) details 101 composite measures of human well-being and progress, covering a broad range of themes from happiness-adjusted income to environmentally-adjusted income, from child development to information and communication technology development.

Even though considerable attention is given to the rankings of composite indicators, many subjective choices are made in their construction: this has motivated studies which perform uncertainty and sensitivity analysis on composite indicator assumptions (Saisana et al., 2005). One important step is the aggregation of indicators, where typically the variables are combined in a weighted average to give the resulting value of the composite indicator. Apart from the decision of which kind of weighted average to use (e.g. arithmetic, geometric), the developer must select values of weights to apply to each variable. The values of these weights can have a large impact on the subsequent rankings, which often goes unnoticed. Understanding the impact of weights on the variation of the composite indicator scores is therefore important.

A possible misconception is that the weight assigned to a variable can be directly interpreted as a measure of importance of the variable to the resulting value of the composite indicator. Indeed, in common approaches to composite indicator weighting such as budget allocation (expert input) and equal weighting, this appears to be the supporting logic. However this is rarely the case: different variances and correlations among variables, for instance, prevent the weights from corresponding to the variables’ importance. Two questions immediately arise: first, given a set of weights and a sample, what is the influence of each variable on the output? Second, how can weights be assigned to reflect the desired importance?

This article puts together, for the first time, tools that allow developers to examine in detail the effect of weights and to subsequently refine the composite indicator. First, building on an earlier proposal of some of the present authors, see Paruolo et al. (2013), the relative importance of each indicator is measured with the *Pearson correlation ratio*, which is a variance-based measure that accounts for (possibly nonlinear) dependence between input variables and the composite indicator (see Section 2). Going beyond Paruolo et al. (2013), who use local-linear regression to estimate the correlation ratio, the main tool used here is Bayesian Gaussian processes, which have the advantage of providing confidence intervals on the Pearson correlation ratio (see Section 3). This overall approach echoes the work of Da Veiga et al. (2009) in the sensitivity analysis literature, but with the additional advantage that it does not require the use of an independent sample from the marginal distribution of each indicator to estimate the variance of the conditional expectation.

As a further step, to better understand the influence of variables on the composite indicator, an approach based on the correlated sensitivity analysis work of Xu and Gertner, 2008a, Xu and Gertner, 2008b is proposed in Section 4, which uses additional regressions to decompose the influence of each variable into influence caused by correlation, and influence caused by the composite indicator structure (aggregation and weights).

Finally, the issue of optimisation of weights is considered in Section 5. Although a linear solution to the optimisation problem is available in Paruolo et al. (2013) using linear regression, the proposal here is to extend it to nonlinear regression, to account for nonlinear main effects. This is done by numerically minimising the difference between correlation ratios and their desired values. Given that this involves a large number of regression fits, penalised splines are preferred over Gaussian processes because of their low computational cost.

The various strands of methodology in this article are demonstrated on three case studies in Section 6, which are chosen as examples related to sustainability and quality of life: the Resource Governance Index, the Good Country Index, and the Water Retention index. The first two indices were chosen because of their potentially high policy impact owing to a considerable media presence. The latter index is chosen for academic purposes, in order to evidence the advantage of using penalised splines in studies with large number of entities (thousands of drainage basins in this case).

The tools to perform these analyses in Matlab are available for free download on the author's web page (Becker, 2017).

## 2 Measures of importance

Consider the case of a composite indicator *y* (output) calculated by aggregating over *d* normalised input variables (indicators) ${\left\{x_{i}\right\}}_{i=1}^{d}$. The most common aggregation scheme is the weighted arithmetic average, i.e.

(1) $$y_{j}=\sum_{i=1}^{d}w_{i}x_{\mathrm{ji}},\;j=1,2,\cdots ,n$$

where *x*_*ji*_ is the normalised score of individual *j* (e.g., country) based on the value *X*_*ji*_ of the *i*th raw variable *i* = 1, …, *d* and $w_{i}$ is the nominal weight assigned to the *i*-th variable, such that $\sum_{i=1}^{d}w_{i}=1$ and $w_{i}>0$.

Now consider an importance measure *I*, which captures the influence of each *x*_*i*_ on *y*, and which is also normalised to sum to one over all *d* variables. The fundamental underlying principle of this paper is that $I_{i}\neq w_{i}$, nor is *I* necessarily linearly related to w, although this fact is sometimes overlooked by developers. In fact, the importance of *x*_*i*_ is also strongly dependent on its (possibly nonlinear) correlations with other variables, which are in turn correlated with each other. Therefore determining and isolating the effect of *x*_*i*_ on *y* is by no means trivial.

For any given composite indicator, one can define measures of importance of each of the input variables *x*_*i*_ with respect to the output *y* of the composite indicator. One approach is to measure the dependence of *y* on *x*_*i*_. Consider the decomposition,

(2) $$y_{j}=f_{i}(x_{\mathrm{ji}})+\varepsilon_{j},$$

where *f*_*i*_(*x*_*ji*_) is an appropriate function, possibly nonlinear, that models the conditional mean of *y* given a sample point *x*_*ji*_—and *ε*_*j*_ is an error term which accounts for variation due to indicators other than *x*_*i*_. A well-known way to measure the *linear* dependence of *y* on *x*_*i*_ is to use the coefficient of determination $R_{i}^{2}$: in sample, this can be computed as:

(3) $${\mathrm{SS}}_{\mathrm{reg},i}/{\mathrm{SS}}_{\mathrm{tot}},$$

where (in the case of *R*^2^) ${\mathrm{SS}}_{\mathrm{reg},i}=\sum_{j=1}^{n}{\left(\hat{f_{i}}(x_{\mathrm{ji}})-\bar{y}\right)}^{2}$ is the sum of squares explained by the *linear* regression, $\bar{y}:=n^{-1}\sum_{j=1}^{n}y_{j}$ is the sample average, $\hat{f_{i}}(x_{\mathrm{ji}})={\hat{\beta }}_{0}+{\hat{\beta }}_{1}x_{\mathrm{ji}}$ is the *linear* fit for observation *y*_*j*_ and ${\mathrm{SS}}_{\mathrm{tot}}=\sum_{j}{\left(y_{j}-\bar{y}\right)}^{2}$ is the total sum of squares. $R_{i}^{2}$ can hence be seen as the ratio of the sum of squares explained by the linear regression of *y* on *x*_*i*_, and the total sum of squares of *y*. Since this measure is based on linear regression, it does not account for any nonlinearities between *y* and *x*_*i*_.

In composite indicators, although the aggregation formula is often linear, a nonlinearity in the relationship between *y* on *x*_*i*_ can be introduced by the correlation between variables. In such cases, $R_{i}^{2}$ may underestimate the degree of dependence. The nonlinear measure adopted here is the *correlation ratio*, *S*_*i*_, *i* = 1, 2, .. ., *d* also widely known as the *first order sensitivity index*, or *main effect index* (see e.g. Becker and Saltelli, 2015). This measure is meant to measure the (possibly nonlinear) influence of each variable on the composite indicator, and is a nonlinear generalisation of $R_{i}^{2}$, such that $R_{i}^{2}$ equals *S*_*i*_ when *f*_*i*_(*x*_*ji*_) is linear. It can be interpreted as the expected variance reduction of the composite indicator scores, if a given variable were fixed. It is defined as:

(4) $$S_{i}\equiv \eta_{i}^{2}:=\frac{V_{x_{i}}\left(E_{{\text{x}}_{∼i}}\left(y|x_{i}\right)\right)}{V\left(y\right)},$$

where **x**_∼*i*_ is the vector containing all the variables (*x*_1_, …, *x*_*d*_) except variable *x*_*i*_. The term $E_{{\text{x}}_{∼i}}\left(y|x_{i}\right)$ is explicitly stated here with its subscript **x**_∼*i*_ to emphasise that it is the expected value of *y* (the composite indicator), at a given value of *x*_*i*_, with the expectation taken over **x**_∼*i*_ (hereafter the subscript *x*_∼*i*_ is omitted to avoid cluttering the notation, such that $E_{{\text{x}}_{∼i}}\left(y|x_{i}\right)\equiv E\left(y|x_{i}\right)$). In other words, it is *conditional on x*_*i*_, e.g. with *x*_*i*_ fixed at one value in its interval of variation. However, $E\left(y|x_{i}\right)$ is specified so that the value that *y* is conditioned on is not specified. Therefore $E\left(y|x_{i}\right)$ is a *function* of *x*_*i*_. It is also known as the *main effect* of *x*_*i*_, and is equivalent to the *f*_*i*_(*x*_*ji*_) discussed previously; therefore it is nothing more than a nonlinear regression fit on a scatter plot of *y* against *x*_*i*_.

Now let $m_{\mathrm{ji}}:={\hat{f}}_{i}(x_{\mathrm{ji}})$, corresponding to the fitted regression value (of *y* on *x*_*i*_) corresponding to the *j*th sample point *y*_*j*_. The correlation ratio *S*_*i*_ can then be estimated in sample as

(5) $${\hat{S}}_{i}=\sum_{j=1}^{n}{\left(m_{\mathrm{ji}}-\bar{m_{i}}\right)}^{2}/\sum_{j=1}^{n}{\left(y_{j}-\bar{y}\right)}^{2}$$

where $\bar{m_{i}}$ is the mean of the *m*_*ji*_, i.e. $\bar{m_{i}}:=n^{-1}\sum_{j=1}^{n}m_{\mathrm{ji}}$, $m_{j}:=\hat{m}(x_{\mathrm{ji}})$. Eq. (5) mimics (3).

In summary, this means that the correlation ratio, which is a nonlinear measure of dependence of *y* on *x*_*i*_, can be estimated simply by fitting a nonlinear regression to a scatter plot of *y* against *x*_*i*_, taking the variance of the resulting curve, and standardising by the unconditional variance of *y*.

## 3 Estimating the main effect

As discussed, the main effect $E\left(y|x_{i}\right)$ is simply the nonlinear regression fit of *y* against *x*_*i*_: therefore it can be estimated by various nonlinear regression methods. This section briefly describes two approaches which are used in this work for their particular advantages. For conciseness, further technical details are left to the references.

### 3.1 Gaussian processes

Gaussian processes (GPs) are recognised as a highly flexible approach to probabilistically modelling nonlinear data, and are widely used in sensitivity analysis (Becker et al., 2011, Becker et al., 2013). GPs are a “probabilistic distribution over functions”, in that a fit through data points is expressed as a random function. The Gaussian process (GP) is based on the assumption that any points *y*_1_, *y*_2_, .. ., *y*_*n*_ are distributed joint-normally, specified by a mean function which returns the mean at any point, and a covariance function which specifies the covariance between any two points as a decreasing function of the distance in each variable *x*_*i*_ between them.

The specification for the GP includes a number of hyperparameters. In this work, a full Bayesian implementation of the GP is used, where prior distributions are assigned to hyperparameters, and the resulting posterior GP distribution is explored using Markov Chain Monte Carlo methods – see Bishop (2006). In this framework the confidence intervals of the GP also include the uncertainty in the estimation of the hyperparameters.

Since the posterior distribution (fitted to the training data) of the GP is explored by MCMC, it is possible to estimate the correlation ratio at each set of hyperparameter values visited in the search (after burn in). Since there are some thousands of MCMC steps, here only 50 samples are taken of posterior distribution to limit computational time, and therefore 50 sample values of the correlation ratio. This information enables the construction of distributions and confidence intervals over *S*_*i*_.

It should be noted that perhaps the main weakness of GPs is that the cost of training scales poorly with *n*, the number of training data, since it involves the inversion of a *n* × *n* covariance matrix at a cost of the order of *n*^3^. For this reason, although GPs are an excellent tool for nonlinear regression (even allowing estimation of correlation ratio in the presence of discontinuities (Becker et al., 2013)), they cannot be applied to large-*n* problems without discarding some of the data points.

Finally, GPs naturally extend into higher dimensions, i.e. multivariate regression. This property is used in the following section to model dependence. In this work, the R package *tgp* is used for full-Bayesian GPs (Gramacy and Taddy, 2010).

### 3.2 Penalised splines

While Gaussian processes are the ideal tool for many of the applications in this study, they encounter difficulties when there are a very large number of data points (see 6.3 or a large number of regressions, as are required in the optimisation of weights (see 5). In such cases, penalised splines offer a fast alternative. Penalised splines are an extension of linear parametric regression (linear in the parameters), but also have the capabilities of nonparametric regression (i.e. local polynomial regression), such as the flexibility to accommodate nonlinear trends in the data (Ruppert et al., 2003). The basis function which is the heart of the spline model is given by ${\left(x-\kappa_{h}\right)}_{+}^{p}$, where the “+” subscript denotes the positive part; in other words, for any number *u*, *u*_+_ = *u* if *u* is positive, and equals zero otherwise. The *κ*_*h*_ parameter is called the “knot” of the basis function. Splines are constructed by using a number *k* = 0, 1, 2, .. ., *K* of *p*-order spline basis functions with different knots *κ*_*h*_, combined with a *p*-order polynomial. In the applications here a value of *p* = 3 is used (cubic splines).

In order to avoid over-fitting, the estimation of the coefficients is constrained to limit the influence of the spline basis terms, resulting in a smoother fit: this results in the *penalised spline* model. The constraining of regression parameters shares similarities with other approaches such as *ridge regression* (see e.g. Hastie et al. (2001)), and *LASSO* (Tibshirani, 1996). The fitting of the resulting smoothing parameter is performed by cross-validation. The fact that penalised splines are only a short step from linear regression means that they can exploit well-known properties to give fast order-*n* algorithms for the calculation of cross-validation measures, therefore the fitting of a penalised spline can be very fast. A further useful property is that penalised splines allow very simple evaluation of derivatives of main effects. This property will be illustrated in Section 6.1.

Finally, note that in the following work “penalised splines” will sometimes be referred to simply as “splines”. All splines used in this investigation are penalised cubic splines.

## 4 Separating correlation from aggregation

It has been widely noted in the sensitivity analysis literature that in the case of correlated inputs, the correlation ratio (first order sensitivity index) *S*_*i*_ captures both the effect of the variable *x*_*i*_ alone, as well as that due to other variables with which it is correlated (Xu and Gertner, 2008a, Xu and Gertner, 2008b, Li et al., 2010, Mara and Tarantola, 2012). Therefore *S*_*i*_ can be decomposed as follows:

(6) $$S_{i}=S_{i}^{u}+S_{i}^{c}$$

where $S_{i}^{u}$ represents the uncorrelated part, and $S_{i}^{c}$ the correlated part. This distinction allows a greater insight into the nature of the influence of the variable. In classical sensitivity analysis, which is concerned with understanding the effects of input uncertainties on the output of a model, the decomposition of the correlation ratio gives further information on how uncertainty might be reduced, since strong correlations imply that variables need to be treated in correlated groups rather than individually. In the context of composite indicators, it can potentially aid the construction and setting of weights: knowing for example that a given variable has $S_{i}^{c}\approx S_{i}$ implies that the value of the weight might have very little impact on the end result, because the impact of the variable is entirely due to its correlation with other variables present in the framework. Further, if $S_{i}^{c}$ results to be negative, this indicates that there is a conceptual problem with the variable. This is caused by negative correlations between variables, which are usually inadvisable in composite indicators; an example of this occurred in the Sustainable Society Index—the conclusion was that in the presence of negative correlations, the indicators should not actually be aggregated and instead presented as a scoreboard (Saisana and Philippas, 2012).

In order to obtain the decomposition in (6), this paper follows Xu and Gertner, 2008a, Xu and Gertner, 2008b, who use a regression-based approach. However, whereas Xu and Gertner assume a linear relationship between *y* and *x*_*i*_, the approach here generalises to associations both between *y* and *x*_*i*_ and between the *x*_*i*_ themselves. The main advantage of the nonlinear regression approach adopted herein is that no prior information about the joint distribution of input variables is required, only a sample from the distribution.

Of the terms in (6), *S*_*i*_ can be readily estimated via a nonlinear regression using one of the regression methods discussed in the previous section. The uncorrelated term $S_{i}^{u}$ can be estimated by a relatively straightforward procedure based on multivariate linear regression. First, the residuals ${\hat{z}}_{i}$ of a regression of *x*_*i*_ on *x*_∼*i*_ are obtained, i.e.,

(7) $${\hat{z}}_{i}=x_{i}-{\hat{x}}_{i}=x_{i}-\left(\beta_{0}+\sum_{l\neq i}^{d}{\hat{\beta }}_{l}x_{l}\right)$$

where the *β*_*l*_ are coefficients of a multivariate linear regression, found by the standard least-squares estimator. This step effectively removes the correlation between *x*_*i*_ and *x*_∼*i*_ by removing the (linear) dependence. Note that this implicitly assumes pairwise linear dependence between the input variables. From this point, the sensitivity of *y* to ${\hat{z}}_{i}$ is estimated in exactly the same way as estimation of *S*_*i*_: a nonlinear regression of *y* on ${\hat{z}}_{i}$ is performed, and the resulting fitted values ${\left\{{\hat{y}}_{j}^{\left(∼i\right)}\right\}}_{j=1}^{n}$ are used to estimate $S_{i}^{u}$ as follows:

(8) $$\sum_{j=1}^{n}{\left({\hat{y}}_{j}^{\left(∼i\right)}-{\bar{y}}^{\left(∼i\right)}\right)}^{2}/\sum_{j=1}^{n}{\left(y_{j}-\bar{y}\right)}^{2}$$

where ${\bar{y}}^{\left(∼i\right)}$ is the mean of the ${\hat{y}}_{j}^{\left(∼i\right)}$. Note that Xu and Gertner use $\bar{y}$ in place of ${\bar{y}}^{\left(∼i\right)}$: while in linear regression the two terms are equal by definition, in nonlinear regression they may differ slightly. For simplicity, only penalised splines are used for the the regression of *y* on ${\hat{z}}_{i}$, however GPs or another approach could also be used.

$S_{i}^{c}$ may now be found simply by recalling that $S_{i}^{c}=S_{i}-S_{i}^{u}$. Note that, if the correlation between variables is negative, it may occur that $S_{i}^{c}$ is negative, and $S_{i}<S_{i}^{u}$. In the case of composite indicators, this would indicate a conceptual problem as discussed.

Finally, if dependence between variables are thought to be nonlinear, the regression of *x*_*i*_ on *x*_∼*i*_ in (7) can also be performed with a nonlinear method. In reality, there is no reason to assume the relationship between the input variables in a composite indicator is necessarily linear. To investigate the difference, multivariate Gaussian process regression is also used in the case studies in this paper (see Section 3.1). A further advantage of this approach is that the dependence need not be pairwise, as the GP is able to model higher-order interactions. However, as discussed, this can impose some limitations on sample size such that samples of around 1000 points or over might become very slow to work with. Still, many composite indicators have sample sizes lower than this limit, and there also exist approaches to apply GPs on large-*N* problems (Rasmussen and Williams, 2006).

In summary, the steps of the methodology described in this section are as follows, repeated for each variable *x*_*i*_:

1. Estimate *S*_*i*_ using a nonlinear regression approach, e.g. GPs or splines (see previous section).
2. Perform a regression of *x*_*i*_ on *x*_∼*i*_. This can be either linear (using multivariate linear regression), or nonlinear (using a multivariate Gaussian process). Denote this fitted regression as ${\hat{x}}_{i}$.
3. Get the residuals of this regression, ${\hat{z}}_{i}$, using ${\hat{z}}_{i}=x_{i}-{\hat{x}}_{i}$.
4. Estimate $S_{i}^{u}$ by a nonlinear regression of *y* on ${\hat{z}}_{i}$, using the same approach as in Step 1.
5. Get $S_{i}^{c}$ by the simple expression $S_{i}^{c}=S_{i}-S_{i}^{u}$.

Notice then that there are three regression steps per variable: a first univariate regression to estimate *S*_*i*_, a second multivariate regression (either linear or nonlinear) to remove the dependence of *x*_*i*_ on *x*_∼*i*_, and a third univariate regression of *y* on the residuals (effectively the “uncorrelated” *x*_*i*_) to obtain $S_{i}^{u}$.

## 5 Optimising weights

Given a set of weights, the tools in the previous sections can be used to analyse in some detail the influence of each variable on the composite indicator. A natural question which subsequently arises is, how can one adjust the weights of the composite indicator in order to achieve the desired influence of each indicator? This question was addressed in Paruolo et al. (2013), in which a unique solution based on linear regression was found. However, when the main effects are nonlinear, more accurate results will be achieved using nonlinear tools.

Here, a simple numerical approach is proposed which searches for the weights that result in the desired importance using an optimisation algorithm. Let ${\tilde{S}}_{i}$ be the normalised correlation ratio of *x*_*i*_, i.e. ${\tilde{S}}_{i}=S_{i}/\sum_{i=1}^{d}S_{i}$. The target normalised correlation ratio is denoted as ${\tilde{S}}_{i}^{*}$: it is assumed that ${\tilde{S}}_{i}^{*}=w_{i}$, i.e. the weights assigned by developers reflect the intended importance. The normalisation of *S*_*i*_ is done only in order to make it directly comparable with the target values (weights), which also sum to one. In Paruolo et al. (2013) the normalisation was done with respect to *S*_1_; in reality any value can be used since we only want to make a relative comparison.

**Fig. 1:**
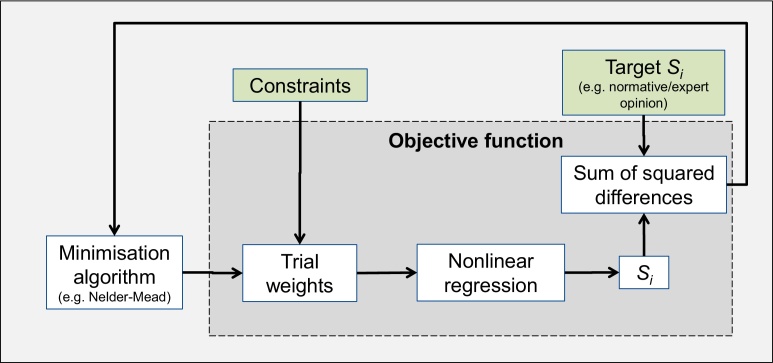
Illustration of the weight-optimisation algorithm.

The optimisation problem is framed by defining an objective function as the sum (over *i* = 1, 2, .. ., *d*) of squared differences between the ${\tilde{S}}_{i}$ at a given set of weights, and the target ${\tilde{S}}_{i}^{*}$. The desired weights $w_{\mathrm{opt}}$ can be found by minimising this objective function, i.e.

(9) $$w_{\mathrm{opt}}={\mathrm{argmin}}_{w}\sum_{i=1}^{d}{\left({\tilde{S}}_{i}^{*}-{\tilde{S}}_{i}(w)\right)}^{2}.$$

where $w={\left\{w_{i}\right\}}_{i=1}^{d}$. Of the many optimisation algorithms available, the approach selected here is the well-known Nelder-Mead simplex search method, since it requires no knowledge of derivatives and tends to produce efficient convergence results (Lagarias et al., 1998). However, if this does not result in a satisfactory solution, other approaches could be used: for an overview on this very large topic of research see e.g. (Nocedal and Wright, 2006). Note that, in contrast to the linear case described in Paruolo et al. (2013), there is no theoretical result that a unique solution exists or that an optimisation algorithm will converge to this solution. However, the success of the procedure can be easily judged by the resulting correlation ratios, and in our test cases a solution has always been found. Fig. 1 shows an overview of the optimisation process applied to the weights.

In practice, the optimised weights may result in negative weights due to the correlation structure between indicators. It is not proposed here that the optimised weights are blindly applied; rather that they are used, among other considerations, as a learning tool to show how far weights are from the “optimal” values, which indicators’ weights have little impact on the results, and even which indicators could be removed. These ideas are illustrated in the following three case studies. Weights can also be used as “scaling coefficients” taking the values 0.5 or 1.0, with the aim of arriving at aggregate scores that are balanced in their underlying components (i.e., that indicators can explain a similar amount of variance in their respective component). This approach has been followed by the developers of the Global Innovation Index since the 2012 edition (Dutta et al., 2014). The use of weights taking the values of 0.5 or 1 is simple enough to be communicated to a larger audience, while at the same time it can ensure statistical soundness in the development of an index.

## 6 Case studies

This section shows the various strands of methodology in the previous section applied to three existing composite indicators. To implement the tools described in the previous sections, codes were written in Matlab. These are available for free download on the author's web page (Becker, 2017), with the exclusion of the Gaussian process estimation of *S*_*i*_ because this involves a complicated link with the R programming language which is not easily transferred to another computer. However, we can supply these codes with instructions on request.

### 6.1 The Resource Governance Index

The Resource Governance Index (RGI) is developed by the Revenue Watch Institute in order to measure the transparency and accountability in the oil, gas and mining sectors in 58 countries (Quiroz and Lintzer, 2013). These nations produce 85 percent of the world's petroleum, 90 percent of diamonds and 80 percent of copper, generating trillions of dollars in annual profits. The future of these countries depends on how well they manage their natural resources.

To evaluate the quality of governance in the extractive sector, the Resource Governance Index employs a 173-item questionnaire, the answers to which are grouped into 45 indicators that are then mapped into three (of the four) RGI dimensions: Institutional and Legal Setting, Reporting Practices, and Safeguards and Quality Controls. The fourth dimension, Enabling Environment, consists of five additional indicators that describe a country's broader governance environment; it uses data compiled from over 30 external sources. The weights are set equal to 0.2, except for Reporting Practices, which is deemed to be twice as important as the other indicators and has a weight of 0.4.

**Fig. 2:**
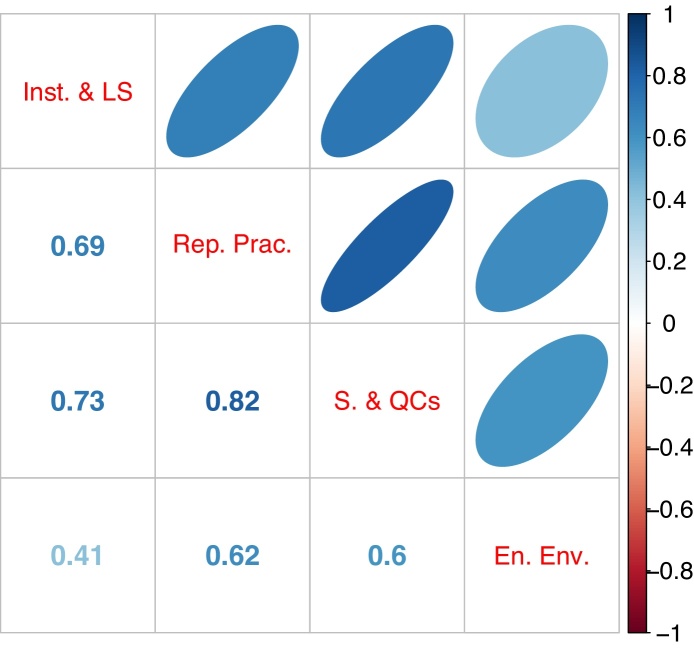
Correlation between variables of the four components of the Resource Governance Index (in order: Institutional and Legal Setting, Reporting Practices, Safeguards and Quality Controls, and Enabling Environment). Numbers represent Pearson correlation coefficients; ellipses represent the strength and direction of the correlation.

**Fig. 3:**
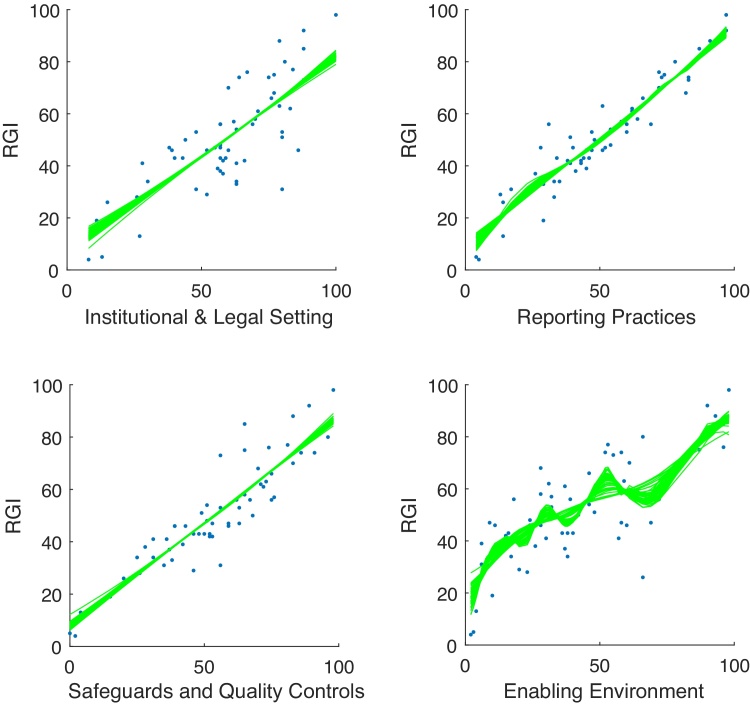
GP main effect fits of the RGI (lines represent samples of the posterior distribution).

**Fig. 4:**
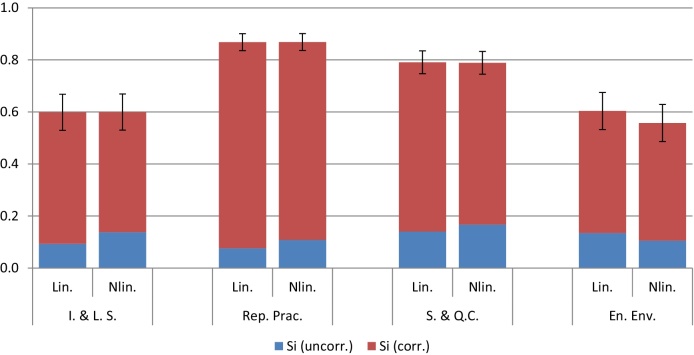
Estimates of *S*_*i*_ (full bars) broken down into $S_{i}^{\left(u\right)}$ (blue) and $S_{i}^{\left(c\right)}$ (red), using both linear and nonlinear dependence modelling. Error bars represent two standard deviations in the estimate of *S*_*i*_, according to GP.

Examining first the correlations between variables, Fig. 2 shows that the four indicators are all correlated with each other positively and with values ranging from 0.82 to 0.41. Fig. 3 shows the GP fits on the RGI data. It is evident that the data is largely linear, but *y* shows some nonlinearity with respect to Enabling Environment. The *S*_*i*_ values, decomposed into correlated and correlated parts, are shown in Fig. 4. Here we must keep in mind that the influence (and therefore the correlation ratio) of Reporting Practices should be twice that of the other three indicators, when clearly it is not (the confidence intervals indicate that there is little uncertainty in these estimate). Instead, its correlation ratio is slightly higher than that of Safeguards and Quality Controls. The other two indicators are less influential, but not by the correct amount. This problem is likely due to the fact that the correlated part of *S*_*i*_ dominates the uncorrelated part in all four indicators, which means that correlations largely dictate influence, rather than weights. A further point is that the linear and nonlinear estimates of $S_{i}^{\left(u\right)}$ are not very different; in this case at least, the linear estimate would have been sufficient.

**Fig. 5:**
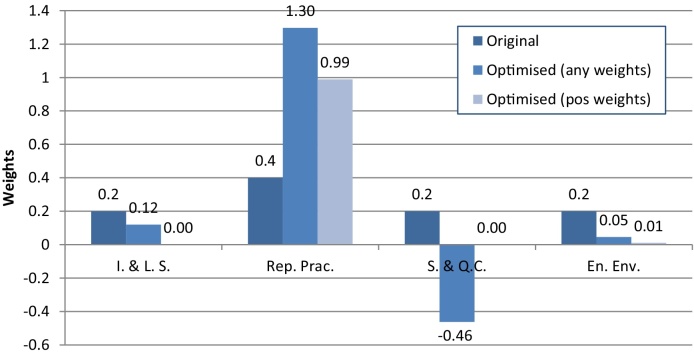
RGI weights for each indicator, showing original weights, optimised weights, and optimised weights with a positive constraint.

Fig. 5 shows the weights for the four components of the RGI—Institutional and Legal Setting, Reporting Practices, Safeguards and Quality Controls, and Enabling Environment—that are obtained through the optimisation procedure; the corresponding normalised *S*_*i*_ values are given in Table 1. The unconstrained optimisation algorithm is able to find weights (0.12, 1.30, −0.46, 0.05) that produce the target correlation ratios, but it requires that the weight of Safeguards and Quality Controls is negative. This is because the original influence of Safeguards and Quality Controls is higher than it should be due to the strong correlations to the other three components. Due to the correlations, it also requires a large change in its weight to give it the desired importance. By constraining the weights to be positive (and to sum to one), the alternative optimised weights offer a slight improvement over the original weights in terms of correlation ratios, but not by a great degree. The influence of Reporting Practices is brought closer to the target, but the influence of the Enabling Environment is slightly further from the target than with the original weights. Moreover, the set of positively-constrained optimised weights results in setting zero weights to three of the RGI components and a weight of almost 1.0 to Reporting Practices.. This is an interesting result in itself, because it suggests that in fact three of the four RGI components could actually be omitted except for Reporting Practices, given that the information about the other components is contained in it through correlations.

**Table 1:** Normalised *S*_*i*_ of the Resource Governance Index. “Original” represents values before weight optimisation; “Optimised” represents values with optimised weights with no constraints; “Optimised (+)” represents values with optimised weights with the constraint that all weights must be positive.

	I. &L. S.	Rep. Prac.	S. &Q.C.	En. Env.
Original	0.209	0.303	0.276	0.211
Optimised	0.200	0.400	0.200	0.200
Optimised (+)	0.187	0.372	0.255	0.185

This case study has served to demonstrate that two strongly correlated variables can hardly have very different influence in the overall index, without resorting to the use of negative weights. By using the tools developed here, these issues become much clearer and allow developers to make more informed decisions.

**Fig. 6:**
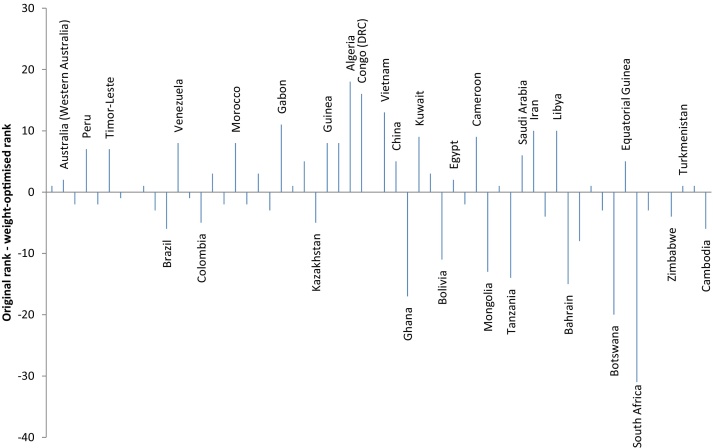
Shifts in rank between original RGI and weight-optimised RGI. Horizontal axis is ordered by weight-optimised rank. Selected countries labelled.

The changes in the RGI ranking as a result of the optimisation of weights (with the constraint that all weights are positive and sum up to one) is shown in Fig. 6. The chart labels the countries with the highest rank changes. Most notably, Algeria improves by 18 places, whilst South Africa drops by 31 places (out of 58). Table 2 also compares the original top ten countries in the ranking with the top ten resulting from optimising weights. The changes are not very large for most countries. Nevertheless, Brazil and Colombia would drop by six and five places, respectively, whilst Peru would gain seven places. The USA becomes the highest ranking country. One should be careful in interpreting these results however: they represent the ranking that would be obtained if “importance” is directly interpreted as nonlinear dependence. Further, they ignore the many other substantial uncertainties involved in the construction of a composite indicator: choices of which indicators to include, how to impute missing data, how to aggregate, and so on. However, one might at least recognise that the rank shifts here represent a credible lower bound on the uncertainty.

**Table 2:** Top ten countries in the RGI: comparison of original against optimised weights. Numbers in brackets refer to the difference between the original rank and the optimised rank.

Rank	Original weights	Optimised Weights
1	Norway (-2)	United States (Gulf of Mexico) (1)
2	United States (Gulf of Mexico) (1)	Australia (Western Australia) (2)
3	United Kingdom (-2)	Norway (-2)
4	Australia (Western Australia) (2)	Peru (7)
5	Brazil (-6)	United Kingdom (-2)
6	Mexico (-1)	Timor-Leste (7)
7	Canada (Alberta) (-3)	Mexico (-1)
8	Chile (0)	Chile (0)
9	Colombia (-5)	Trinidad and Tobago (1)
10	Trinidad and Tobago (1)	Canada (Alberta) (-3)

### 6.2 The Good Country Index

The Good Country Index (GCI) is developed by the Good Country Party with a view to measure what a country contributes to the common good of humanity, and what it takes away (Anholt and Govers, 2014), according to its developers’ normative framework and world view. In total, 125 countries are included in the Index. In contrast to the majority of similar composite indicators, the Good Country Index does not measure what countries do at home; rather, it aims to start a global discussion about how countries can balance their duty to their own citizens with their responsibility to the wider world.

The GCI builds upon 35 indicators that are produced by the United Nations and other international agencies, and a few by NGOs and other organisations. Most of the indicators used are direct measurements of world-friendly or world-unfriendly behaviour (such as signing of international treaties, pollution, acts of terrorism, wars) and some are rather indirect (such as Nobel prizes, exports of scientific journals). The 35 indicators are split in seven components of five indicators each. These seven components, which closely mirror the dimensions of the United Nations Charter, are: Science, Technology & Knowledge; Culture; International Peace and Security; World Order; Planet and Climate; Prosperity and Equality; and Health and Wellbeing. A ranking is calculated for each of the seven components. The Good Country Index is then calculated by taking the arithmetic average of the seven ranks. All seven components are weighted equally with a weight of 1/7.

**Fig. 7:**
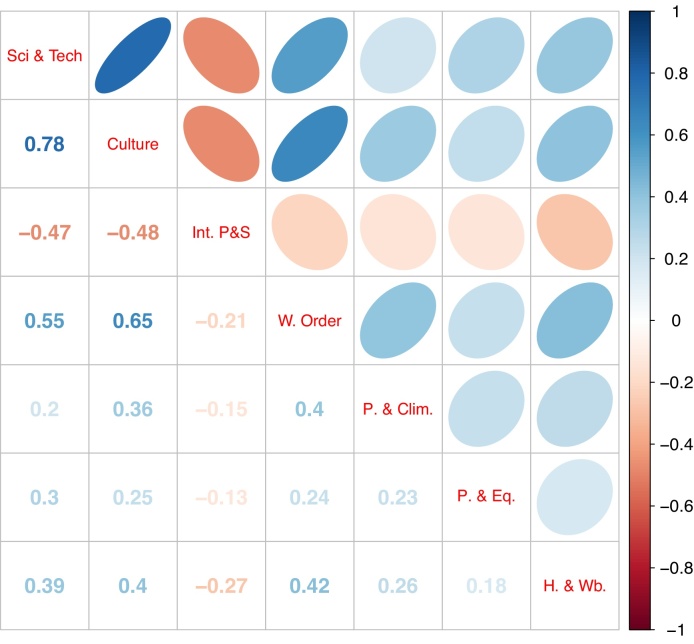
Correlation between the seven components of the GCI (in order: Science, Technology & Knowledge; Culture; International Peace and Security; World Order; Planet and Climate; Prosperity and Equality; and Health and Wellbeing). Numbers represent Pearson correlation coefficients; ellipses represent the strength and direction of the correlation.

**Fig. 8:**
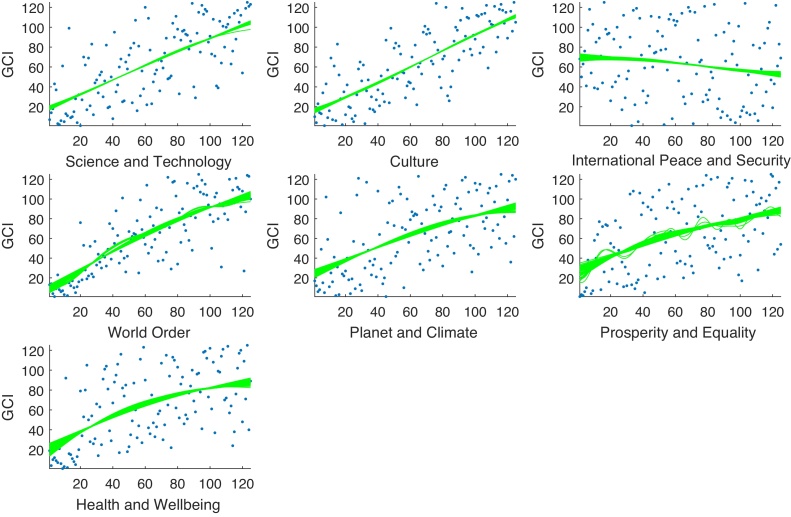
GP main effect fits of the GCI (lines represent samples of the posterior distribution).

**Fig. 9:**
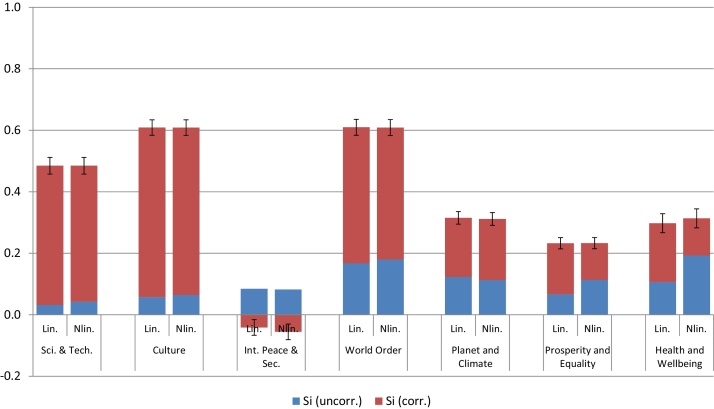
Estimates of *S*_*i*_ (full bars) broken down into $S_{i}^{\left(u\right)}$ (blue) and $S_{i}^{\left(c\right)}$ (red), using both linear and nonlinear dependence modelling. Error bars represent two standard deviations in the estimate of *S*_*i*_, according to GP.).

Examining the GP fits (see Fig. 8) it is evident that while six of the seven components have a positive and fairly linear effect on the GCI, International Peace and Security has a slightly negative main effect. This is further reflected in Fig. 9, which shows the very low effect of International Peace and Security on the composite. In fact, the correlated part of *S*_*i*_ is actually negative—as discussed earlier this implies a problem with this component because, as is evident from Fig. 7, it is negatively correlated with the six remaining components (see Fig. 7). A further conclusion from Fig. 9 is that three components—Planet and Climate, Prosperity and Equality, Health and Wellbeing—have relatively low *S*_*i*_ values.

**Fig. 10:**
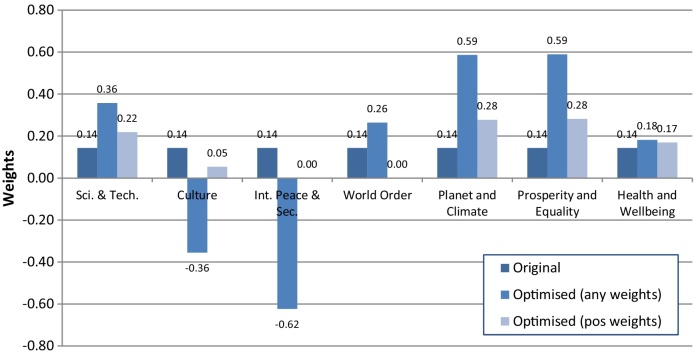
GCI weights for each indicator, showing original weights, optimised weights, and optimised weights with a positive constraint.

The results of the (unconstrained) optimisation of weights are shown in Fig. 10. The most notable result here is that the weight of International Peace and Security is required to be negative due to the strong and negative correlations to other GCI components. The weight of Culture is also required to be negative, for the reason that its effect must be reduced. Indeed, Culture has the strongest bivariate correlations to other GCI components (0.78 with Science and Technology, and 0.65 with World Order), hence it needs a large change in its weight to properly adjust its effect. This problem is further evidenced by the fact that Culture has the largest $S_{i}^{\left(c\right)}$ of all GCI components.

Table 3 shows that under the constraint that the weights are positive, the influence of each component is fairly equal, apart from International Peace and Security, which is somewhat less than the others. Moreover, Culture, International Peace and Security, and World Order, all have zero or near-zero weights. This shows that, if one is prepared to accept a slightly lessened influence of International Peace and Security, one could remove the three components completely, and the result would be a fairly well-balanced composite indicator which still captures the influence of the removed components simply by correlation.

**Table 3:** Normalised *S*_*i*_ of the GCI. “Orig.” represents values before weight optimisation; “Opt.” represents values with optimised weights with no constraints; “Opt. (+)” represents values with optimised weights with the constraint that all weights must be positive.

	S&T.	Cult.	I.P. &S.	W.O.	P. &C.	P. &Eq.	H. &Wb.
Orig.	0.187	0.235	0.017	0.235	0.122	0.090	0.115
Opt.	0.143	0.143	0.143	0.143	0.143	0.143	0.143
Opt. (+)	0.162	0.159	0.055	0.163	0.149	0.152	0.159

**Fig. 11:**
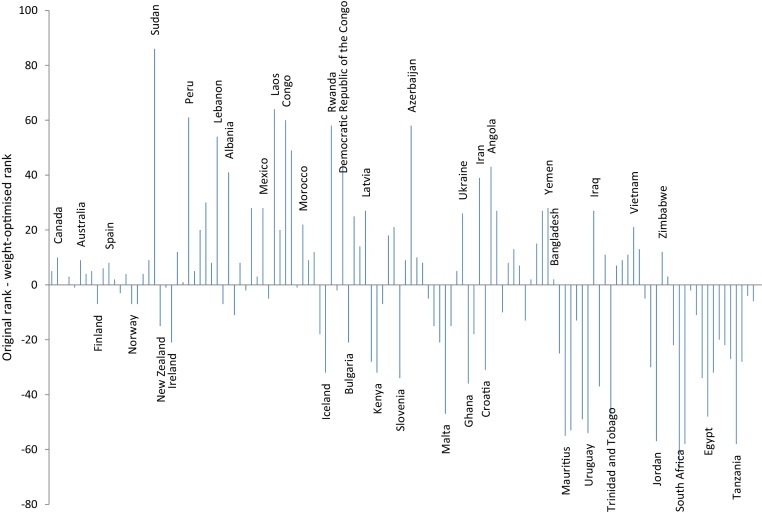
Shifts in rank between original GCI and weight-optimised GCI. Horizontal axis is ordered by weight-optimised rank. Selected countries labelled.

The changes resulting from the optimisation of weights are shown in Fig. 11. Most notably Sudan improves by 86 places (out of 125 countries). Instead, Ireland drops from first place to 21st. The largest decline is experienced by South Africa, which loses 67 places. The comparison of the top ten performers before and after optimisation in Table 4 shows that New Zealand loses 15 places, going from 5th to 20th place, while Canada and Australia enter the top ten with gains of ten and nine places respectively.

**Table 4:** Top ten countries in the GCI: comparison of original against optimised weights. Numbers in brackets refer to the difference between the original rank and the optimised rank.

Rank	Original weights	Optimised Weights
1	Ireland (-21)	Sweden (5)
2	Finland (-7)	Canada (10)
3	Switzerland (0)	Switzerland (0)
4	Netherlands (-1)	United Kingdom (3)
5	New Zealand (-15)	Netherlands (-1)
6	Sweden (5)	Australia (9)
7	United Kingdom (3)	France (4)
8	Norway (-7)	Germany (5)
9	Denmark (-7)	Finland (-7)
10	Belgium (-3)	Luxembourg (6)

### 6.3 The Water Retention Index

The Water Retention Index (WRI) was developed to assess the potential of a landscape to regulate the quantity of water passing through it (Vandecasteele et al., 2016). The index reflects the relative capacity for water retention, and is used as a tool to assess the impact of land management scenarios at the European scale. It uses several data sets that are directly affected by changes in land use, allowing it to be re-calculated based on future land use projections.

The index is comprised of four parameters that take into account the physical potential for water retention in the landscape. These include interception by vegetation (Rv), storage in surface water bodies (*R*_*wb*_), soil moisture (*R*_*s*_), and percolation to groundwater stores ($R_{g}w$). In addition, the influence of average slope and soil sealing are taken into account. The first four parameters and the slope together describe the physical capacity of the land to retain water, therefore reflecting the maximal potential retention of water in the natural environment. Lastly, the influence of artificially sealed areas are taken into account as a multiplication factor to compute the final index, representing the actual potential for water retention.

**Fig. 12:**
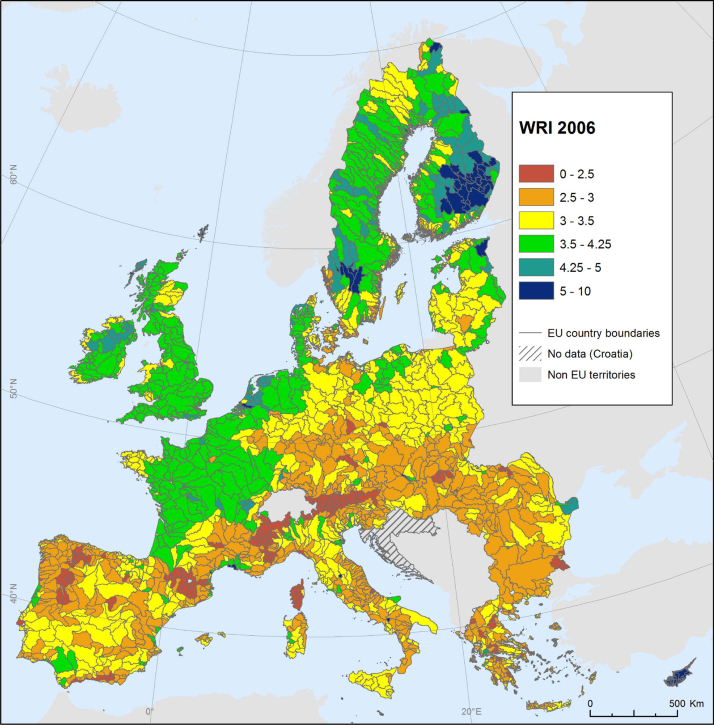
Water retention index as calculated for 2006 at the catchment level.

The WRI is different from the previous two case studies in that it can be calculated at resolutions as high as the 1km^2^ level, across Europe. However, the most relevant resolution for policy-making is to evaluate the index at the level of catchment areas (drainage basins)—see Fig. 12. Across Europe, even this lower resolution results in a data set 4789 points, each with indicator values and resulting WRI values. This makes it somewhat challenging from a computational point of view, in terms of estimating the correlation ratio and optimising the weights to reflect the desired importance. Here, the advantages of penalised splines come to the fore: since they can be fitted very efficiently, they bring such large-*n* composite indicators within reach of the tools described here.

**Fig. 13:**
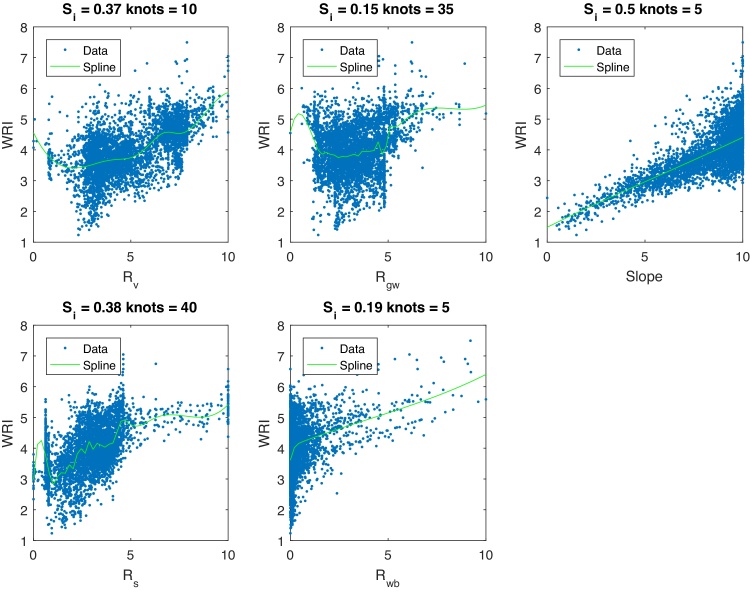
Spline fits to WRI data prior to optimisation.

The indicators in the WRI are only weakly correlated; for this reason the correlated and uncorrelated correlation ratios are not presented here. Fig. 13 shows the spline fits to the WRI data. There are clearly strong skews in each indicator, and significantly nonlinear responses. In this case, linear regression would not be an adequate tool. Table 5 shows the results of the weight-optimisation algorithm using penalised splines. Of the five indicators, it is clear that the normalised *S*_*i*_ do not reflect the desired equal importance, with the Slope variable having three times the influence of *R*_*gw*_, for example. Using the optimisation algorithm, the optimised weights constrained to positive values result in all five indicators having equal importance to the overall index.. This is largely due to the fact that there are no strong correlations between indicators, so weights do not need to be adjusted by large margins, as is the case with the previous case studies (RGI and GCI).

**Table 5:** Weights and normalised *S*_*i*_ of the Water Retention Index. “Original” represents values before weight optimisation; “Opt” represents optimised weights with no constraints.

	Weights		Normalised *S*_*i*_	
	Original	Opt	Original	Opt
$R_{v}$	0.200	0.170	0.231	0.200
*R*_*gw*_	0.200	0.290	0.096	0.200
Slope	0.200	0.100	0.313	0.200
*R*_*s*_	0.200	0.140	0.238	0.200
*R*_*wb*_	0.200	0.280	0.122	0.200

## 7 Conclusions

For developers and users of composite indicators, three exploratory tools have been presented herein which together allow for a detailed analysis of the importance of weights assigned to the index components. First, one can determine the true influence of each component to the overall index via the correlation ratio, which accounts for nonlinearity in the data associations. This value comes with confidence intervals if the estimation follows a Bayesian Gaussian process. Second, one can decompose the correlation ratio into a part caused by correlations with other index components, and a part caused purely by the indicator itself. This decomposition allows one to identify indicators whose influence in the overall index is not greatly affected by their weight value (i.e. those indicators with a high correlated Si compared to the uncorrelated part), and also whether correlations are responsible for negative effects, among other things. This information does not emerge by examining the correlation matrix alone. Third, one can estimate the values for the weights that would lead to the desired importance of the index components. The tools proposed here could also generalise to composite indicators with a large sample size (a few thousand of data points) by the use of penalised splines. The statistical methods used here are coded in Matlab and available to download as a package at (Becker, 2017).
